# Rheumatoid Arthritis-Linked Artificial Joint Infections Leading to Amputations

**DOI:** 10.7759/cureus.35622

**Published:** 2023-02-28

**Authors:** Laurence Stolzenberg, Austin Huang, Mohammad Usman, Alexis Koch, John Stevenson, Colby Kihara, Jason Seale

**Affiliations:** 1 Orthopedic Surgery, Alabama College of Osteopathic Medicine, Dothan, USA; 2 Neurology, Alabama College of Osteopathic Medicine, Dothan, USA; 3 Anesthesiology, Alabama College of Osteopathic Medicine, Dothan, USA; 4 Internal Medicine, Alabama College of Osteopathic Medicine, Dothan, USA; 5 Gastroenterology, Alabama College of Osteopathic Medicine, Dothan, USA; 6 Medicine, Alabama College of Osteopathic Medicine, Dothan, USA; 7 General Surgery, Decatur Morgan Hospital, Decatur, USA

**Keywords:** sulfasalazine, amputation, joint infections, immune suppressing medications, orthopedics, orthopedic surgery, prosthetic joint infection, rheumatoid arthritis

## Abstract

Rheumatoid arthritis (RA) is a common autoimmune condition that can rarely cause more serious complications, such as permanent joint damage or infection, and may pose a significant additional risk during certain routine procedures. One major consequence of RA is that it can lead to serious and permanent joint damage requiring arthroplasty. Additionally, RA is a known cause of infection, with orthopedic prosthetic joint infections (PJIs) being documented. We explore one such serious case of a patient with long-term RA and a left knee joint replacement who presented to the emergency room with a serious PJI. History revealed that he repeatedly was affected by infections and had an extensive and severe clinical course, including nine revision surgeries. After a physical examination, imaging was performed, which further supported the diagnosis of joint infection. Considering the extensive number of attempts to salvage the joint, clinicians decided an above-knee amputation was necessary. This case highlights the fact that RA both increases the need for orthopedic arthroplasties and the risk of complications from these procedures, complicating clinical decision-making for physicians. Additionally, this patient had other underlying medical conditions and social habits that may have contributed to his severe clinical course, and we hope to explore these, discuss possible methods of modifying them, and assist clinicians in not only better treating similar patients but also emphasizing the importance of further developing standardized predictive algorithms and scoring tools.

## Introduction

Rheumatoid arthritis (RA) is an autoimmune disease that causes chronic, systemic inflammation, which can lead to inflammatory arthritis as well as extra-articular involvement [1]. The etiology of RA is not fully understood; however, various alleles have been identified that have a strong association with developing the condition [2-4]. RA primarily affects synovial joints, and these affected joints are often in a symmetric distribution. Progression of joint involvement often begins in small peripheral joints and then proceeds to involve large joints, such as knees [1]. RA patients will often present with swelling, arthralgias, erythema, decreased range of motion, and stiffness that is worse in the morning upon waking up and classically improved by evening [5,6]. Appropriate management of RA is essential as those affected can have a significantly decreased quality of life and increased risk for infections, cardiovascular disease, cancer, osteoporosis, respiratory disease, and mortality than the general population [5]. There is no known cure for RA; however, management is aimed at suppressing the activity and severity of the disease. Treatment with short-term nonsteroidal anti-inflammatory drugs (NSAIDs) and corticosteroids has been shown to decrease pain and stiffness in those affected by RA [7]. It is important to note, however, that these analgesic and anti-inflammatory medications do not improve or slow down disease progression, but instead improve the overall quality of life [6]. Other various long-term disease-modifying antirheumatic drugs (DMARDs) are other options that, as the name implies, modify the disease course.

Infections are of major concern for those afflicted with RA as they contribute to increased overall mortality [8-12]. Associations between RA and infections have been well documented in the literature, including various cohort studies that have found an increased risk of serious infections in RA patients [8,13]. However, there is no one specific etiology responsible for the pathogenesis of these infections, rather it is likely to occur due to a multifactorial set of issues. Infections can also be the result of the disease itself due to immobility and immunological dysfunction [14]. The hypothesis behind the immunological dysfunction resulting from RA is that premature aging of the immune system may result in a weakened ability to fight off pathogens [15].

Other major contributors to immunological dysfunction in patients with RA are chronic comorbid conditions, such as diabetes mellitus (DM) and kidney disease, as well as social factors such as smoking [11,15]. Smoking has been shown to have a significant impact on the immune system, which promotes infection. Inhalation of the chemicals in cigarette smoke results in the formation of oxidative moieties that activate immune cells such as macrophages to secrete proinflammatory cytokines [16]. Additionally, cigarette smoke can affect immune system signaling pathways and modify transcription, which can result in immunosuppression and anti-inflammatory effects [16]. The result of this spectrum of possible effects from cigarette smoke determines the overall modification of the immune system [16]. 

Finally, the increased risk of infection may be due to treatments such as joint surgery or pharmacological treatments such as DMARDs, immunosuppressants, and steroids [14,15,17]. Joint surgery in RA has been shown to increase the development of septic arthritis [18,19]. Glucocorticoids are the mainstay of RA treatment and have been well researched and have been linked to increased risk of major infections in several rheumatic diseases [20].

As RA progresses, joints can often become severely deformed, thus having a significant impact on the patient's quality of life. One option available to patients is joint replacement. Joint replacement is a procedure to improve the patient's quality of life with overall benefits such as reducing pain and restoring independence [21]. Rates of joint surgeries, including joint arthroplasties, have been rising and are expected to continue to increase [21,22]. Whether or not a prosthetic joint is involved, joint infection is an inherent complication of any joint surgery. Specifically, with prosthetic joint infections (PJIs), comorbid conditions such as obesity and DM can compound this risk for infections [23-31]. Of note, RA has been found in multiple studies to be associated with an increased risk of PJIs [23,27,28,32-37]. Other risk factors have also been documented such as male gender, smoking, arthroplasty revision surgery, and antecedent septic arthritis of the specific joint [22,26,27,30-32,36-44]. 

The pathogenesis of postoperative joint infections is thought to be due to the introduction of microorganisms during surgery. However, the contiguous and hematogenous spread of microorganisms can also result in joint seeding. The most common infectious organisms are gram-positive cocci, most of which are Staphylococcus aureus and coagulase-negative staphylococci [30,32,45-56]. Patients with joint infections often present with pain, joint swelling or effusion, erythema, warmth, fever, drainage, and/or the presence of a sinus tract communicating with the arthroplasty [45,57-59]. The diagnosis is made with consideration of clinical, laboratory, and radiological findings with intraoperative inspection and tissue evaluation [21]. Treatment is often a combination of both antibiotics and surgical intervention [21].

## Case presentation

This patient was a 72-year-old male with multiple underlying medical and social conditions, including RA, tobacco abuse, hypertension, and a history of opioid abuse who presented to the emergency department with a complaint of left lower extremity (LLE) swelling, redness, and purulent drainage for the last four weeks. He had been taking oral doxycycline as prescribed by his family physician for five days, but his symptoms had continued to worsen. The patient was admitted to integumentary abnormalities, swelling, and an ulcerated lesion on his LLE but denied any other symptoms. Family history was significant for RA in both parents, and the patient endorsed an allergy to intravenous (IV) contrast. A review of home medications was notable for the previously mentioned doxycycline, sulfasalazine 1,500 mg twice daily, ibuprofen 800 mg thrice daily, and hydroxychloroquine 200 mg twice daily. Physical examination revealed unremarkable respiratory, cardiovascular, and gastrointestinal examinations. When attention was turned to the LLE, swelling, fluctuance, warmth, and tenderness over the left knee were noted. The left dorsalis pedis pulse was noted as diminished, being graded as 1+. The patient was noted to have a normal gait. The patient was fully alert, oriented, and in no acute distress, although he was described as ill-appearing. Vitals were within normal ranges with a temperature of 97.5 °F, a pulse of 85 beats per minute, a body mass index (BMI) of 20.5 kg/m^2^, a respiratory rate of 20 breaths per minute, and a blood pressure of 154/86 mmHg.

**Figure 1:**
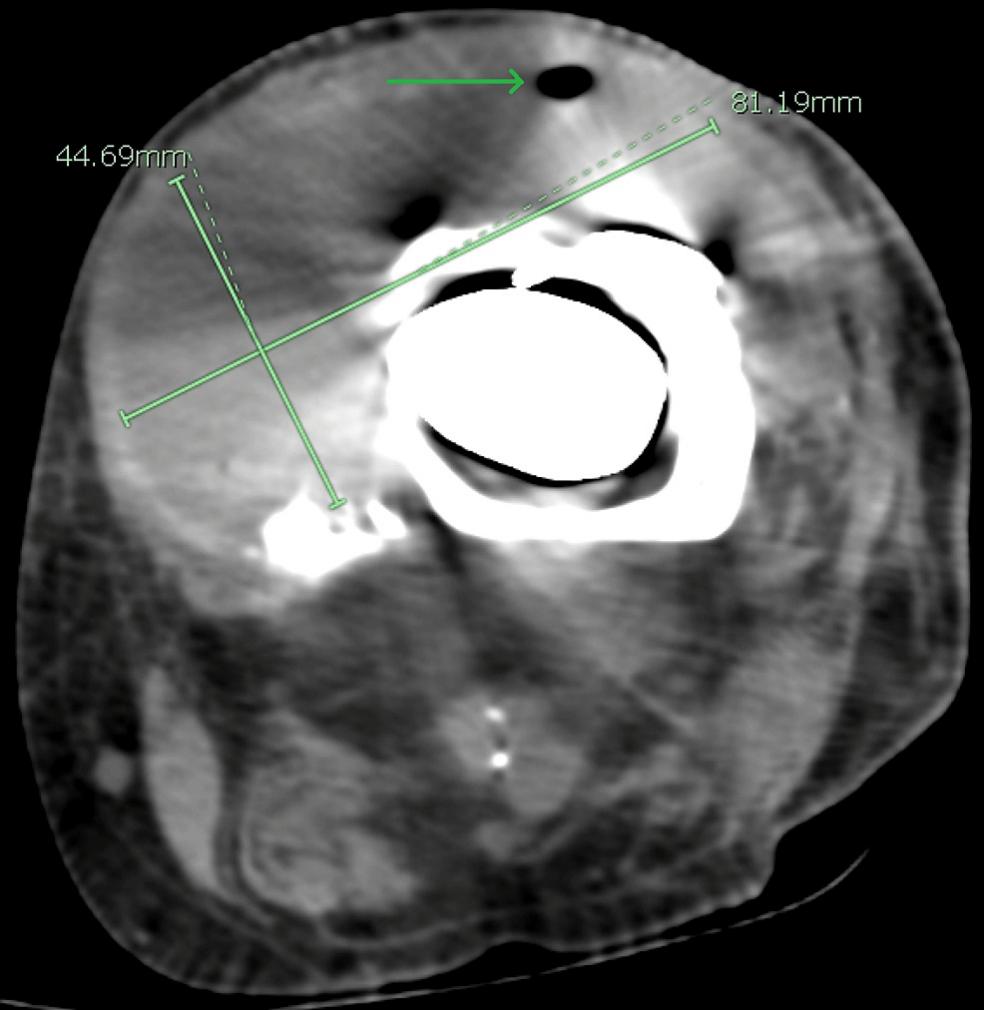
CT of the left lower extremity, transverse cut, showing gas bubbles (green arrow) in the fluid collection, suggestive of an abscess. CT, computed tomography

**Figure 2:**
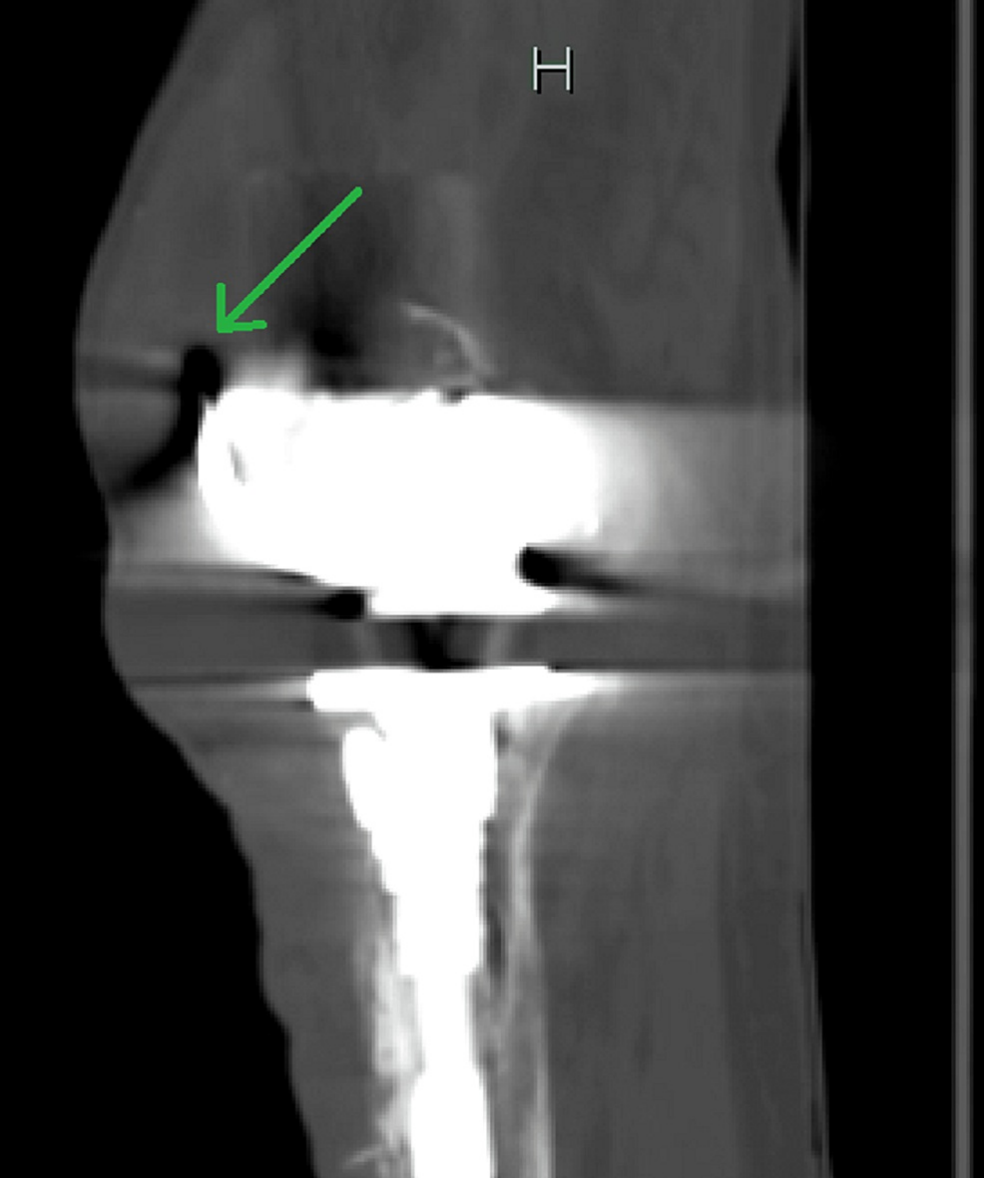
CT of the left lower extremity, sagittal cut, showing gas bubbles (green arrow) in the fluid collection, suggestive of an abscess. CT, computed tomography

Initial laboratory investigations included a complete metabolic panel (CMP), which was unremarkable, liver function tests within normal limits, and renal function tests (RFTs), which were equally unremarkable with a blood urea nitrogen (BUN) of 21 mg/dL and serum creatinine (sCr) of 0.8 mg/dL. Glucose was 91 mg/dL, and serum calcium was at the upper end of normal, revealing a value of 10.2 mg/dL. Complete blood count (CBC) was remarkable for a significantly elevated white cell count of 28,110 cells/µL, a low hemoglobin level of 12.6 mg/dL, and a low hematocrit of 37%. A urinalysis was also completed and found to be normal as well. Imaging performed included computed tomography (CT) of the lower extremities, which showed a left knee arthroplasty with a long stem prosthesis. These images also revealed a collection of fluid with internal droplets of gas anterior to the knee measuring 8.1 cm × 4.5 cm × 12.3 cm, suggesting an abscess (Figures 1-2). The patient was empirically placed on vancomycin, piperacillin-tazobactam, and morphine. 

The patient was then admitted to the hospital where surgery and orthopedics were consulted for further management. The hospitalist obtained further history, which revealed an extensive number of surgeries on the LLE, beginning with a total left knee arthroplasty in 2014. He said it got infected and replaced in 2015 with a Teflon implant. In 2017, there was a second infection, which was treated with two separate instances of antibiotic spacer placement and another replacement of the prosthetic joint. He endorsed a further infection in 2018 where surgery was performed to cut nonviable muscle and place a graft. In total, the patient endorsed having had nine separate surgical procedures on his LLE, with the most recent one in 2018. The last surgeon installed an antibiotic spacer, which was considered a final attempt at saving the limb before considering above-knee amputation. Repeat physical examination revealed the possibility to express grossly purulent drainage from a small ulcer in the lateral portion of the left knee as well as a large soft tissue fluid collection anterior to the joint. Vancomycin was continued, but piperacillin-tazobactam was discontinued and replaced with cefepime based on hospital protocol.

The general and orthopedic surgeons decided an urgent above-knee amputation was necessary, and it was scheduled for the following day. During surgery, a large pocket of pus was encountered anterior to the joint, as delineated by imaging, while separating the soft tissue and collected for culture. The bone had a *moth-eaten* appearance, and debridement of the medullary cavity of the femur was required with a curette. The surgery was performed by both the general and orthopedic surgeons with no complications other than an estimated blood loss of 50 mL.

The patient recovered from surgery relatively well, although treating pain while minimizing opioid use was challenging. The patient was also affected by severe anxiety and anorexia, which were treated successfully with dronabinol. Multiple cultures from the fluid collected during surgery were inconclusive, but empiric antibiotic therapy was continued. On postsurgery day 9, the patient was discharged home with outpatient follow-up with infectious disease, orthopedics, general surgery, and home health. He was set up with a mechanical wheelchair and appropriate pain measures with minimal opioids, considering his history of opioid dependence and personal preference to minimize opioids and oral antibiotics. He is expected to do well as of the time of this paper.

## Discussion

This patient underwent multiple extensive surgeries in an attempt to resolve the recurrent severe infection in his prosthetic knee, and as such, it is reasonable to assume that this patient was affected greatly. Not only did he have to deal with significant levels of pain, which might have contributed to his opioid abuse and dependence, but his condition significantly affected his quality of life and activities of daily living. As such, the considerable morbidity experienced by this patient implores us to further explore the underlying causes of his recurrent infections and discover if any other treatments could have been considered to improve his condition. It is unfortunately quite difficult to pinpoint the exact origin of his multiple subsequent infections, but we will attempt to go over the possible etiologies one by one. The first condition to consider is his RA. While it is difficult to isolate the specific level of risk due to that disease process alone, multiple studies have cited this condition as a significant risk factor [23,27,28,32-37]. Aside from RA, it is also important to explore other social habits, risk factors, and medications that likely contributed to the development and progression of his infections.

Our patient was a heavy smoker, with a history of smoking half a pack a day for 63 years. Literature has established a link between smoking and generally increased risk of infection and thus likely played a role in his clinical course [15,27,60,61]. Although it is nearly certain that many physicians and healthcare professionals suggested and counseled them to quit smoking, it would perhaps be worth considering if anyone pointed out the well-supported link between these infections and tobacco use to the patient [15,27,60,61]. During his stay in the hospital, he had been able to temporarily refrain from smoking with help from a nicotine patch, so it is reasonable to suppose further pharmacological therapy could have at least assisted him in this difficult endeavor.

Considering our patient’s severe clinical course, we need to review comorbid conditions that may have contributed to increased infection risk. This patient denied any history of DM, which was supported by his blood glucose levels being consistently within normal limits. His estimated glomerular filtration rate (eGFR) also precluded any form of chronic kidney disease. A commonly cited risk factor for the development of PJIs is obesity. In this specific case, our patient was on the other end of the spectrum, with a BMI of 20.5 kg/m^2^. One study found that a low BMI (<25 kg/m^2^) was associated with an increased risk of PJIs, possibly due to a low nutritional reserve and immunosuppression [62]. Interestingly, treating malnutrition as a possible intervention for PJI prevention has been a recent area of research interest, with multiple pharmaceutical interventions showing promise in treating this risk factor [61]. In this case, dronabinol successfully improved this patient’s anorexia. Dronabinol use is an area of research that has received increasing attention, and there have been some promising data showing that its use may benefit patients with chronic malnutrition [63,64]. However, more research is certainly needed to conclude whether this patient or similar patients would benefit from regular outpatient dronabinol use. 

Nonmodifiable risk factors, such as male sex and advanced age likely also played a role in the pathogenesis of this patient’s recurrent infections. It is widely documented in the existing literature that advanced age can increase the risk of specific kinds of infections, and indeed skin and soft tissue infection rates are elevated in the elderly. Further complicating the management of this patient is the fact that nosocomial infections are a major risk factor in the elderly. In fact, not only are elderly patients more likely to spend lengthy amounts of time in a hospital setting, putting them at risk, but they are also more likely to become infected each day they stay in the hospital [65]. Another nonmodifiable intrinsic risk factor in this patient is that he is of the male sex, which has specifically been shown to increase the risk of PJIs [26,37-41].

An additional risk factor for this patient is his multiple revision surgeries. Research has shown that the incidence of PJIs is higher in patients that have undergone an arthroplasty revision surgery when compared to primary implantation [30-32,36,42-44,66]. This is likely due to prolonged operating time. Our patient had undergone extensive repair surgeries following multiple joint infections, with each surgery increasing his risk for a future infection. There are possibly other reasons for the increased risk associated with revision surgeries that could be covered in further work.

Medications are important to review as many medications are known to cause immunosuppression or increase the risk of infection, and they can be modified to mitigate risk. The patient took ibuprofen regularly. Regular ibuprofen use does not seem linked to any increase in the risk of infection as a review of available literature demonstrates no strong or significant association between NSAIDs and PJIs. Multiple publications support the benefits of NSAIDs during joint infections, prosthetic or not [67,68]. Next, this patient took sulfasalazine daily. While an increased risk of infection is noted in the documentation associated with this medication [69], no published case reports specifically linking this medication to infections were found when using multiple keywords. Additionally, the information available on this medication from the American Academy of Rheumatologists has no mention of infections [70]. Finally, hydroxychloroquine revealed a similar pattern of contraindication and reported infections in existing literature as sulfasalazine [71]. Existing literature specifically mentions these are the safest agents for use in the perioperative management of an RA patient. A review of the literature shows many other treatments for RA are associated with higher risks of infection [72], although these were not included in his treatment plan. Based on current guidelines and an in-depth review of medication, we can state that the management of this patient’s pharmaceutical regimen followed all the latest guidelines and literature, and it is doubtful that using the other available agents would have prevented his complications.

When considering this patient’s severe and prolonged clinical course with PJIs, discussed risk factors, and active medications, it is important to consider if an initial arthroplasty should have been performed at all. While clinical acumen is important in these decisions, the numerous factors described above and the overall complex clinical picture show the need for a standardized method to aid clinical decision-making. Recently, there has been a push to develop a tool to aid in the assessment of the risk of developing PJIs. Of note, the Mayo PJI risk score (Mayo Clinic, Scottsdale, AZ, USA) is one such tool; however, it is not yet fully validated [62]. The Mayo PJI risk score accounts for multiple risk factors such as BMI, prior joint surgery, immunosuppression, American Society of Anesthesiology (ASA) score, and procedure duration to assist in risk stratification and consider additional preventative measures. We believe further work on this scoring tool would be of great assistance to patients and may help prevent severe clinical courses as seen in this patient.

## Conclusions

We hope this case will help future clinicians in managing similar patients affected by both autoimmune disorders such as RA and repeated infections to improve clinical outcomes for those undergoing arthroplasties. As the rate of procedures such as arthroplasties rises in the elderly population, so does the risk of complications, notably infections. As such, clinicians should consider all underlying conditions that could cause significant complications when planning such procedures and during the postoperative care environment. Clinicians should potentially consider interventions such as further or more potent prophylactic antibiotics, more aggressive treatment of the underlying RA, or more radical interventions such as above-knee amputations and the use of a prosthetic limb in the future if presented with a similar patient affected by repeated infections. It is also important to analyze the risk and benefits before planning potentially complicated procedures. In addition, if it is decided that further arthroplasties are indicated, potentially using a different surgical technique or different replacement parts intraoperatively should be considered. We also hope this case will assist other clinicians working on predictive models and algorithms to help score the likelihood of PJIs before arthroplasty.
